# Linarin Enriched Extract Attenuates Liver Injury and Inflammation Induced by High-Fat High-Cholesterol Diet in Rats

**DOI:** 10.1155/2017/4701570

**Published:** 2017-06-27

**Authors:** Zhen-Jie Zhuang, Chao-Wen Shan, Bo Li, Min-Xia Pang, Han Wang, Yan Luo, Yin-lan Liu, Yu Song, Nan-Nan Wang, Su-Hong Chen, Jun-ping Shi, Gui-Yuan Lv

**Affiliations:** ^1^Zhejiang Chinese Medical University, Hangzhou, Zhejiang 310053, China; ^2^The Affiliated Hospital of Hangzhou Normal University, Zhejiang 310015, China; ^3^Hangzhou Normal University, Hangzhou, Zhejiang 3110036, China; ^4^Wenzhou Medical University, Wenzhou, Zhejiang 325035, China

## Abstract

The aim of this study was to explore the potential beneficial effects of linarin enriched Flos Chrysanthemi extract (Lin-extract) on nonalcoholic steatohepatitis (NASH) induced by high-fat high-cholesterol (HFHC) diet in rats. SD rats received normal diet, HFHC diet, or HFHC diet plus different doses of Lin-extract. The liver content of triglyceride and total cholesterol markedly increased in HFHC diet-fed model rats while middle and high dose of Lin-extract lowered liver cholesterol significantly. The expression of stearoyl-CoA desaturase (SCD1) was upregulated by HFHC diet and further elevated by high dose Lin-extract. High dose of Lin-extract also markedly lowered the serum alanine aminotransferase (ALT) and aspartate aminotransferase (AST) and inhibited the activation of c-Jun N-terminal kinase (JNK) induced by HFHC in livers. The HFHC-increased mRNA levels of hepatic inflammation cytokines, including monocyte chemotactic protein-1 (MCP-1), tumor necrosis factor-*α* (TNF-*α*), and chemokine (C-X-C motif) ligand 1 (CXCL1), were suppressed by Lin-extract dose-dependently. Furthermore, pathology evaluation showed that high dose Lin-extract greatly improved lobular inflammation. Our results suggest that Lin-extract could attenuate liver injury and inflammation induced by HFHC diet in rats. Its modulatory effect on lipid metabolism may partially contribute to this protective effect.

## 1. Introduction

As life style, especially dietary style change, the incidence of metabolic syndrome is rising. The manifestation of metabolic syndrome in liver is nonalcoholic fatty liver disease (NAFLD), which is mainly caused by western diet which is rich in saturated fat and cholesterol [1]. Twenty-five to thirty percent of NAFLD patient may develop nonalcoholic steatohepatitis (NASH), which is closely associated with diabetes, hypertension, and other metabolic diseases, and may even develop hepatocarcinoma (HCC) [2, 3]. In fact, HCC has already become the primary cause of obesity-related cancer death in middle-aged men in the USA [4]. As the hepatitis B is now effectively prevented by vaccination, NASH associated HCC will become the major cause of liver cancer in China in future. Therefore, it is essentially important to prevent the transition from simple steatosis to NASH.

The classic theory “two hits” was proposed to explain the mechanism of this transition. Increased uptake of fatty acids and de novo lipogenesis and/or reduced fatty acid oxidation and very low density lipoprotein (VLDL) secretion lead to excess lipid accumulation in the liver, which sensitized liver to further injury such as oxidative stress, ER stress, and activated c-Jun N-terminal kinase (JNK) signaling pathway [5]. Challenged hepatocytes release danger signal and then trigger the activation of Kupffer cells and infiltration of other immune cells, leading to the release of inflammatory mediators such as interleukin-1*β* (IL-1*β*) and tumor necrosis factor-*α* (TNF-*α*) [6]. In recent years, growing evidence suggested that triglyceride (TG) per se is not the culprit of lipotoxicity, while free fatty acids especially saturated ones and cholesterol are the chief criminals to cause injury [7]. On the other hand, unsaturated fatty acids have opposite effects and may favor the formation of lipid droplet. In fact, TG protects hepatocytes from damage caused by free fatty acids (FFAs) by sequestering them inside lipid droplets [8–10].

*Flos Chrysanthemi Indici* (FCI), the flower of* Chrysanthemum indicum* L., is a commonly used herb in traditional Chinese medicine with antimicrobial, antioxidative, and antimycotic properties [11–14]. Linarin (LIN), a flavonoid compound rich in FCI, has been shown to exert various pharmacological effects, including anti-inflammatory, neuroprotective, cardioprotective, and antioxidative effects [15–18]. We previously showed that Lin-extract from FCI has antihypertension effect in a spontaneous hypertensive rat model [19]. Since hypertension is very closely associated with obesity, NASH, and other metabolic syndrome, we test the possibility of this extract in preventing HFHC diet induced liver injury and explored the potential mechanism of its bioactivity.

## 2. Material and Methods

### 2.1. Chemicals, Diet and Reagents

Linarin extract was processed from* Flos Chrysanthemi* (Zhejiang Chinese Medical University, Traditional Chinese Medicine Decoction Pieces, Ltd). The amount of linarin was determined by HPLC analysis as described in our previous protocol [19]. The linarin content in the extract is 72%. HFHC diet (10% fat, 1% cholesterol, 0.2% cholate) were prepared by TROPHIC Animal Feed High Tech Co. (Nantong, China). Kits for serum biochemistry analysis including cholesterol (TC), triglyceride (TG), high density lipoprotein-cholesterol, (HDL-c), low density lipoprotein-cholesterol (LDL-c), glucose and alanine transaminases (ALT), and aspartate transaminases (AST) were purchased from MeiKang Chemical Co. (Ningbo, China). Liver TC and TG quantification kits were from Applygen Technologies Inc. (Beijing, China). Both First-strand cDNA synthesis kit and FastStart Universal SYBR Green Master (ROX) kit were products from Roche (USA). BCA kit for protein quantification was from Beyotime Biotechnology (Shanghai, China). Phospho-SAPK/JNK (Thr183/Tyr185) and SAPK/JNK primary antibody were purchased from Cell Signaling Technology (USA).

### 2.2. Animal Study

Male Sprague–Dawley rats (180–200 g) were purchased from Shanghai Experimental Animal Center of Chinese Academy of Sciences and kept in 22 ± 1°C on a 12 h light/dark cycle. All animals were allowed to acclimatize for one week on the normal diet and then were randomly divided into five groups (*n* = 10 each group): control group was fed with a normal chow diet, model group received a HFHC diet, and intervention groups were given a HFHC diet and at the mean time given orally linarin enriched extract (Lin-extract) at the dose of 15 mg/kg, 30 mg/kg, and 60 mg/kg, respectively. After 4-week feeding, animals were fasted overnight, with exsanguinations under light pentobarbital. Blood was drawn from the ophthalmic venous plexus for biochemistry analysis. Livers were quickly removed and weighed. Same parts of liver in each rat were snap-frozen in liquid nitrogen for RNA, protein study, and oil red O staining. Small pieces of liver were fixed in neutral-buffered formalin for histology examination. Animal handling and sample collection were conducted in accordance with Use and Care of Laboratory Animals published by the Zhejiang Province (2009).

### 2.3. Serum Biochemistry

Blood was centrifuged at 3000 rpm for 10 min. The supernatant was collected and the level of TG, TC, LDL-c, HDL-c, and glucose and the activity of ALT and AST were measured by TBA-40FR automatic clinical chemistry analyzer (Toshiba, Japan).

### 2.4. Liver TG and TC Quantification

The quantification of TC and TG was performed following the instruction of commercial kit from Applygen Technologies Inc. Equal amounts of frozen liver tissue were homogenized in lysis buffer and incubated at room temperature for 10 minutes. 5 *μ*l supernatant was taken for protein quantification. Additional 200 *μ*l of supernatant was heated in 70°C for 10 minutes and then centrifuged at 2000*g* for 5 minutes. 20 *μ*l of supernatant was used as substrate to incubate with R1 and R2 reagent, allowing the formation of product based on GPO Trinder reaction, which brought absorption change catalyzed by an enzyme at 550 nm. OD was measured by plate reader (Biorad, USA) and TC and TG concentration was determined by standard curve and further normalized by protein concentration.

### 2.5. HE and Oil Red Staining

For light microscopic analysis of liver histology, the paraffin-embedded liver tissues were cut into 5 *μ*m sections, and standard hematoxylin-eosin (HE) staining procedure was performed. Ten light microscopic fields were viewed on each section and the pattern of steatosis and number of inflammation foci were evaluated. For oil red O staining, frozen blocks were cut into 10 *μ*m sections, fixed with formalin for 10 minutes, and then stained with oil red reagent for 15 minutes. After washing with PBS for 3 times, nucleus was counterstained with hematoxylin.

### 2.6. Quantitative RT-PCR

Total RNA was extracted from frozen liver tissue with Trizol reagent. Equal amount of isolated RNA was reverse transcribed into cDNA using first-strand cDNA synthesis kit. Real-time PCR was performed using a FastStart Universal SYBR Green Master (ROX) kit on an ABI7900 HT thermal cycler (Applied Biosystems, Carlsbad, CA) according to the manufacturer's instructions. GAPDH was used as reference gene and results were analyzed by ΔΔCt method. Sequences of the primers are listed in Table 1.

### 2.7. Western Blotting

Frozen liver tissues were homogenized in cold 1x RIPA buffer [10 mmol/L Tris-HCl (pH 8.0), 140 mmol/L NaCl, 1 mmol/L EDTA (pH 8.0), 0.5 mmol/L EGTA, 1% Triton X-100, 0.1% SDS, and 0.1% sodium deoxycholate] containing phosphatase inhibitors and 1 mmol/L PMSF. The homogenate was incubated on ice for 10 minutes and centrifuged at 15,000*g* for 10 minutes. The protein concentration in supernatant was determined with BCA kit and equal amount of protein was loaded onto SDS-PAGE and subject to electrophoresis. Proteins were transferred onto a PVDF membrane. The membrane was blotted with 5% BSA for one hour, incubated with phospho-SAPK/JNK (Thr183/Tyr185) and SAPK/JNK primary antibody at 4°C overnight. Primary antibody was removed by washing the membrane with TBST (0.1% tween 20) for 3 times and 15 minutes each time. Second antibody conjugated to horseradish peroxidase (HRP) was blotted with membrane at room temperature for one hour; then the membrane was washed again as done with primary antibody and developed using enhanced chemiluminescence HRP substrate. Images of the membranes were taken with a Micro Chemi System (Israel).

### 2.8. Statistics Analysis

Data were expressed as means ± SEM unless indicated otherwise. Difference among means was analyzed using One-way ANOVA followed by LSD test in SPSS 17. *p* < 0.05 was considered statistically significant. For lobular inflammation analysis, Mann–Whitney test in SPSS 17 was employed to compare each intervention group with model group, respectively.

## 3. Results

### 3.1. Effect of Lin-Extract on Serum Biochemical Profile

As shown in Table 2, HFHC diet induced markedly increase of the serum levels of TC and LDL-c and decreased the HDL-c levels. Lin-extract reversed this profile change weakly but consistently at different dosages, even though the differences did not reach statistical significance.

### 3.2. Effect of Lin-Extract on Liver Index and Liver Lipid Metabolism

**Figure 1:**
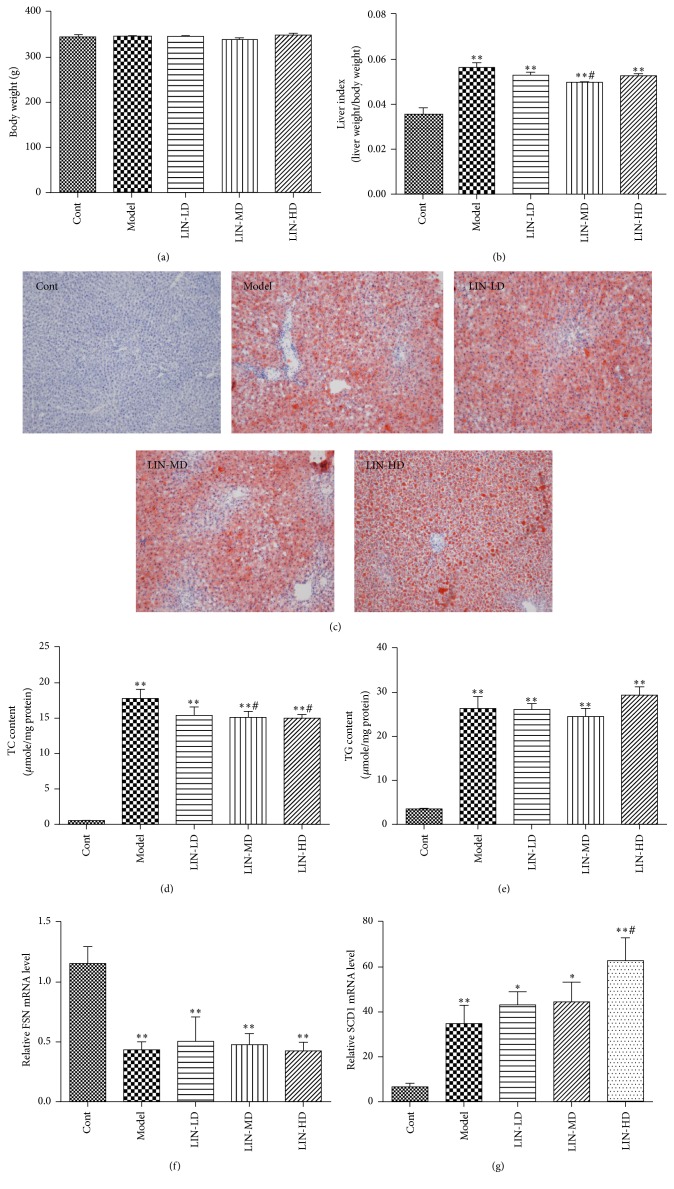
Regulatory effect of Lin-extract on liver index and liver lipid metabolism. (a) Body weight and (b) liver index comparison among different groups. Values are expressed as the mean ± SEM (*n* = 10) (^*∗∗*^*p* < 0.01, control versus model group). (c) Oil red O staining of liver section at 100x magnification. ((d) and (e)) Quantification of total glyceride and total cholesterol content in the liver. Values are expressed as the mean ± SEM (*n* = 10) (^*∗*^*p* < 0.05, ^*∗∗*^*p* < 0.01 control versus model group). (f) FASN and (g) SCD1 gene expression by real-time PCR in liver. Values are expressed as the mean ± SEM (*n* = 10) (^*∗*^*p* < 0.05 control versus model group, ^#^*p* < 0.05 high dose versus model group). Cont, control; LIN-LD, low dose Lin-extract; LIN-MD, middle dose Lin-extract; LIN-HD, high dose Lin-extract; FASN, fatty acid synthase; SCD1, stearoyl-CoA desaturase 1.

No body weight difference was observed among all groups (Figure 1(a)). Liver index (the ratio of liver weight to body weight) was calculated to reflect the extent of hepatomegaly. High-fat high-cholesterol (HFHC) diet-fed rats in model group had higher liver index than those in control group (5.6%  ± 0.082 in model group versus 3.6%  ± 0.084 in control group, *p* < 0.01); Lin-extract decreased liver index in all three intervention groups as compared with model group, although significance existed only in middle dose group (*p* < 0.05) (Figure 1(b)). In model group and Lin-extract treated group, liver sections showed strong oil red O staining while control group showed negative staining. The TG and TC content in the rat livers were further quantified with commercial kits. Results show that TG and TC increased dramatically in model group comparing to control; middle and high dose Lin-extract significantly lowered the TC content in the liver. TG content was not significantly affected by low and middle dose Lin-extract and even subtly increased in high dose Lin-extract group comparing to model group (Figures 1(c)–1(e)). Quantitative RT-PCR was used to examine the effect of Lin-extract on genes related to lipid metabolism. Results showed that mRNA levels of fatty acid synthase (FASN) were downregulated in model group and intervention groups comparing to control group, which indicated de novo synthesis of fatty acid was suppressed to adapt to excess exogenous lipid intake. Interestingly, the expression of stearoyl-CoA desaturase 1 (SCD1), which catalyzes the conversion of saturated fatty acid to unsaturated fatty acid was upregulated in model group, which may reflect the fact that the body tried to accommodate the toxic saturated acid by converting them into unsaturated fatty acid. High dose Lin-extract enhanced this protective defence mechanism by further upregulating the expression of this enzyme (Figures 1(f) and 1(g)).

### 3.3. Lin-Extract Alleviated the Liver Injury Induced by HFHC Diet

**Figure 2:**
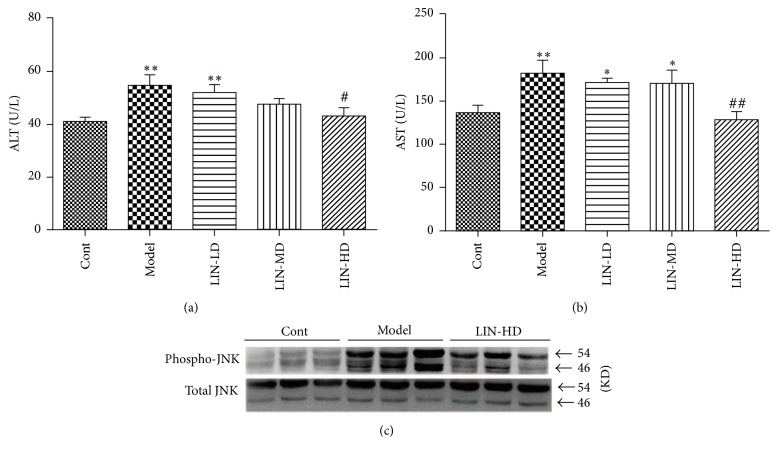
Lin-extract alleviated liver injury by JNK signaling pathway. Lin-extract lowered the release of liver enzymes (a) ALT and (b) AST. Values are expressed as the mean ± SEM (*n* = 10) (^*∗*^*p* < 0.05, control versus model, and ^*∗∗*^*p* < 0.01, control versus model; ^#^*p* < 0.05 and ^##^*p* < 0.01, model versus intervention group). (c) Lin-extract inhibited the activation of JNK signaling pathway. 3 representative samples from control, model, and Lin-HD group were presented. Cont, control; LIN-LD, low dose Lin-extract; LIN-MD, middle dose Lin-extract; LIN-HD, high dose Lin-extract; ALT, alanine aminotransferase; AST, aspartate aminotransferase.

HFHC diet-fed rats in model group had significantly higher levels of serum ALT and AST than those fed with normal diet in control group. Lin-extract dose-dependently decreased HFHC diet-elevated serum ALT and AST levels. High dose Lin-extract treatment lowered the ALT from 54.90 ± 12.31 U/L in model group to 43.20 ± 9.86 U/L in high dose group (*p* < 0.05) and AST from 182.40 ± 45.87 U/L in model group to 128.10 ± 30.97 U/L in high dose group (*p* < 0.01) (Figures 2(a) and 2(b)). The excess fatty acid led to oxidative stress and ER stress, which activated JNK signaling pathway. Results of western blot showed JNK was highly phosphorylated in model group and the phosphorylation was inhibited by high dose Lin-extract (Figure 2(c)), suggesting Lin-extract alleviated the lipotoxicity-induced liver injury.

### 3.4. Lin-Extract Improves Liver Inflammation Induced by HFHC Diet

**Figure 3:**
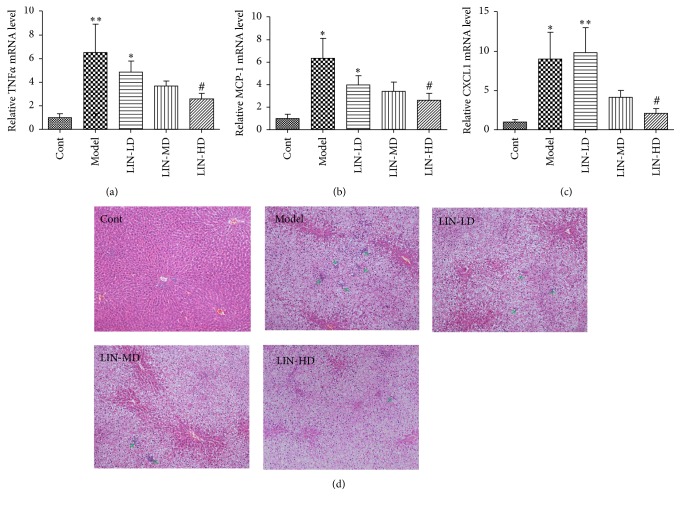
Lin-extract improved liver inflammation induced by HFHC diet. Lin-extract dose-dependently suppressed the expression of inflammation cytokines (a) TNF-*α*, (b) MCP-1, and (c) CXCL1. Values are expressed as the mean ± SEM (*n* = 10). (^*∗*^*p* < 0.05, control versus model; ^#^*p* < 0.05, model versus intervention group). Lin-extract improved inflammation infiltration. (d) HE staining in representative sections at 100x magnification. Green arrow points to inflammation foci. Cont, control; LIN-LD, low dose Lin-extract; LIN-MD, middle dose Lin-extract; LIN-HD, high dose Lin-extract; TNF-*α*, tumor necrosis factor-*α*; MCP-1, monocyte chemotactic protein-1; and CXCL1, chemokine (C-X-C motif) ligand 1. ^*∗∗*^*p* < 0.01, control versus model.

To examine the potential anti-inflammation effect of Lin-extract, the expression of several inflammation cytokines was measured by real-time PCR. Monocyte chemotactic protein-1 (MCP-1), tumor necrosis factor-*α* (TNF-*α*), and chemokine (C-X-C motif) ligand 1 (CXCL1) were dramatically upregulated by HFHC diet and suppressed dose-dependently by Lin-extract treatment (*p* < 0.05, *p* trend < 0.05, Figures 3(a)–3(c)). HE staining showed absence of steatosis and inflammation in control group. Both model group and Lin-extract treated group displayed diffused steatosis, which was predominantly microvesicular. Many central veins collapsed presumably due to the pressure from enlarged hepatocytes. The lipid droplets present in >90% hepatocytes per field of view. The severity of inflammation was graded based on Kleiner and Brunt criteria. The grade distribution was listed in Table 3. The model group showed the most severe inflammation with 2 out of 9 reaching grade 3; the maximum inflammation foci in one 200x field are as high as 10. The inflammation is significantly improved by Lin-extract at high dose (*p* < 0.05) (Figure 3(d)).

## 4. Discussion

In this study, we reported that Lin-extract showed mild and favorable regulatory effect on lipid metabolism and could efficiently alleviate the liver injury and inflammation induced by HFHC diet without improving steatosis.

The NASH model we used in this study is induced by high-fat high-cholesterol diet. In this model, steatosis and lobular inflammation were rapidly induced in 4 weeks. There is also very mild fibrosis in 2 out of 10 rats at this time point. In our model, we did not observe obvious body weight increase comparing to control group, but the liver index increased dramatically. With similar diet formula, but continued for 9 weeks, Ichimura et al. reported slower body weight gain in HFHC diet group than normal diet group [1]. In fact, NASH without obesity was also observed in clinic; approximately one-third of patients with NAFLD have a nonobese phenotype, especially those in Asia [20, 21]. Therefore, this model is meaningful in studying NASH treatment.

In current NASH model, we discovered that Lin-extract can regulate the cholesterol level in both blood and liver. All doses of Lin-extract treatment slightly decreased serum total cholesterol and LDL-c and increased HDL-c. In the liver, total cholesterol was significantly downregulated by middle and high dose Lin-extract. Recent studies strongly suggest cholesterol plays an important role in converting simple steatosis into NASH [1, 22]. Ichimura et al. compared the pathology progression of NASH induced by high-fat diet and high-fat plus 1.25% or 2.5% cholesterol diet and demonstrated that cholesterol dramatically increased the TG content in the liver and accelerated the progression of NASH. Rats fed with high-fat diet plus cholesterol in their study developed severe NASH in only 9 weeks with fibrosis. Therefore, one of the mechanisms of Lin-extract inhibiting NASH might be through lowering cholesterol.

By studying the lipogenesis related gene, we found that FASN dramatically downregulated in all HFHC diet groups, while the expression of SCD1 increased in model groups. We interestingly found that SCD1 was further upregulated in livers of rat fed with high dose Lin-extract comparing to model group. SCD is the rate-limiting enzyme in the synthesis of monounsaturated fatty acids (MUFA) from saturated fatty acid (SFA). TG levels increase in cells exposed to both MUFA and SFA. However, the TG accumulation in MUFA treated cells is 2-fold more than other cells [23]. MUFA have been demonstrated to prevent saturated fatty acid-induced ER stress, inflammation, apoptosis, and insulin resistance in hepatocytes and skeletal cells [24, 25]. The favorable effect might be due to its lipogenic effect [26, 27]. This suggests that the slight increase of TG in the liver of high dose group in our study may be due to the elevated MUFA production catalyzed by increased SCD1. Consistent with our study, increased SCD1 expression in the liver has been reported in a study of mice fed with high-fat diet treated with rosiglitazone [28]. In addition, Oosterveer et al. observed that the SCD1 expression increased after treatment with fenofibrate accompanied with increased hepatic TG [29]. Another study by Shiri-Sverdlov et al. observed fenofibrate treatment inhibiting NASH induced by high-fat diet in ApoE−/− mice [29, 30]. Recent research indicated that TG is protective to NASH by requesting toxic free fatty acid and free cholesterol in the droplet. This may explain, with the same or even higher lipid burden, that the liver shows less injury evidenced by lower ALT and AST levels and weaker JNK pathway activation. Lin-extract may alleviate HFHC diet induced stress by altering the balance between saturated fatty acid and monounsaturated fatty acid through upregulation of SCD1 expression. Flavonoids and polyphenols from Chinese herb or food such as hawthorn leaf flavonoids, quercetin, and lotus root polyphenolic extract have been reported to lower steatosis by downregulating lipogenesis through SREBP1c or by enhancing *β*-oxidation pathway [31–33]. It seems not to be the case with Lin-extract in our study. It might be due to the difference in animal model used, or linarin indeed modulates lipid metabolism in different mechanism as fenofibrate does.

We found Lin-extract also suppresses inflammation in the liver. This might be the consequence of the optimization of lipid composition. It may also be due to its anti-inflammation nature. The main ingredient of the extract, linarin, is a flavonoid. Flavonoid's anti-inflammation properties have been demonstrated in numerous studies [34, 35]. One of the anti-inflammation mechanisms is through inhibiting the secretion and expression of proinflammatory factors [35]. For example, the activation of NF-*κ*B and release of TNF-*α* and IL-6 in human monocytes in response to hyperglycemia were suppressed by 3–10 mM luteolin [36]. Quercetin attenuated the high-fat diet induced pancreatic histopathological damage and reduced the mRNA and protein expression of NF-*κ*B, IL-1*β*, IL-6, and TNF-*α* [37]. It was reported that linarin suppressed LPS-induced NO release in macrophage cell line RAW264.7 [17]. The activation of Kupffer cell, the macrophage in the liver, is the essential step mediating NASH [38]. Linarin also showed strong anti-inflammation activity in carrageenan-induced rat paw edema model [39]. In a D-galactosamine and LPS-induced fulminant hepatic failure model, Kim et al. reported that linarin could attenuate the elevated serum and hepatic expression of TNF-*α*, IL-6, and IFN-*γ* [40]. In addition, several studies suggested linarin had anticholinesterase (AchE) activity [41, 42]. AchE is the enzyme which hydrolyzes acetylcholine and inhibition of AchE activity will increase the level of acetylcholine. The latter not only is an important neurotransmitter, but also the mediator of cholinergic anti-inflammation pathway [7]. Neostigmine, an AchE inhibitor, has been shown to suppress the elevated expression of proinflammation cytokines in an acute liver failure model. Zhou et al. reported that activating 7 nicotinic acetylcholine receptor, the mediator of cholinergic anti-inflammation pathway, suppressed the release of TNF-*α* and IL-6 in a mouse NASH model. Therefore, another potential mechanism of anti-inflammation effect by Lin-extract may be related to its anti-AchE activity from linarin, although this hypothesis needs to be proved in the future study.

In this study, we provide the first report of the beneficial effect of Lin-extract in liver disease induced by HFHC diet. Combining this extract with another lipid-lowering drug may be a promising strategy in the treatment of NASH or other NASH associated disease. We have to admit that there are some limitations in our study. The medication used here is an extract; the biological function may also come from ingredients other than linarin in the extract. The second issue is the animal model we used. The cholesterol content in the diet is relatively high; animal developed severe NASH in relatively short time; this may limit the detection of the cholesterol lowering effect from Lin-extract. Milder and more close to clinical situation model in long term treatment should be explored.

## Figures and Tables

**Table 1:** Primer sequences for real-time polymerase chain reaction.

Gene	Forward primer (5′ to 3′)	Reverse primer (5′ to 3′)
TNF-*α*	ATGGGCTCCCTCTCATCAGT	GCTTGGTGGTTTGCTACGAC
MCP-1	TAGCATCCACGTGCTGTCTC	CAGCCGACTCATTGGGATCA
GAPDH	GGCACAGTCAAGGCTGAGAATG	ATGGTGGTGAAGACGCCAGTA
FSN	AAGGACCTGTCTAGGTTTGATGC	TGGCTTCATAGGTGACTTCCA
SCD1	TCTAGCTCCTATACCACCACCA	TCGTCTCCAACTTATCTCCTCC
CXCL1	AGACAGTGGCAGGGATTCAC	GGGGACACCCTTTAGCAGCATCT

**Table 2:** Comparison of serum biochemistry profile in different groups (*n* = 10, $\overset{-}{x}\pm s$).

Group	Control	Model	LIN-LD	LIN-MD	LIN-HD
TG (mmol/L)	1.00 ± 0.29	1.05 ± 0.27	1.00 ± 0.19	1.09 ± 0.30	0.96 ± 0.22
TC (mmol/L)	1.98 ± 0.26	5.15 ± 1.36^*∗∗*^	4.65 ± 1.40^*∗∗*^	4.40 ± 1.00^*∗∗*^	4.72 ± 1.04^*∗∗*^
HDL-c (mmol/L)	0.96 ± 0.12	0.88 ± 0.09^*∗∗*^	0.92 ± 0.12	0.90 ± 0.09	0.85 ± 0.12^*∗∗*^
GLU (mmol/L)	3.83 ± 0.59	3.91 ± 1.23	3.73 ± 0.78	3.23 ± 0.76^*∗*^	3.95 ± 0.54
LDL-c (mmol/L)	0.82 ± 0.14	4.06 ± 1.35^*∗∗*^	3.53 ± 1.44^*∗∗*^	3.28 ± 0.97^*∗∗*^	3.68 ± 1.03^*∗∗*^

^*∗*^ *p* < 0.05, and ^*∗∗*^*p* < 0.01 compared with normal control group. Values are means ± SD. LIN-LD, low dose Lin-extract; LIN-MD, middle dose Lin-extract; LIN-HD, high dose Lin-extract; TG, triglyceride; TC, total cholesterol; HDL-c, high density lipoprotein-cholesterol; LDL-c, low density lipoprotein-cholesterol; Glu, glucose.

**Table 3:** Lobular inflammation score in different group based on Kleiner and Brunt criteria.

Lobular inflammation score	Control (*n* = 10)	Model (*n* = 9)	LIN-LD (*n* = 10)	LIN-MD (*n* = 10)	LIN-HD (*n* = 10)
0 (no foci)	10	—	—	2	2
1 (<2 foci)	—	2	2	3	5
2 (2–4 foci)	—	5	7	5	3
3 (>4 foci)	—	2	1	—	—

LIN-LD, low dose Lin-extract; LIN-MD, middle dose Lin-extract; LIN-HD, high dose Lin-extract.
